# Obesity as a Confounding Factor in the Diagnosis of Wilson’s Disease: Case Report of Two Siblings with the Same Genotype but Different Clinical Courses

**DOI:** 10.3390/cimb46060365

**Published:** 2024-06-17

**Authors:** Emanuele Bracciamà, Annamaria Sapuppo, Laura Rapisarda, Enrico Siciliano, Anna Caciotti, Amelia Morrone, Martino Ruggieri, Giuseppina Cantarella, Renato Bernardini, Gaetano Bertino

**Affiliations:** 1 Hepatology Unit, Department of Clinical and Experimental Medicine, Policlinico “G. Rodolico—San Marco” Hospital, University of Catania, 95123 Catania, Italy; emanuele.bracciama@gmail.com (E.B.); sicilianoenrico92@gmail.com (E.S.); gaetanobertinounict@gmail.com (G.B.); 2 Pediatric Clinic, Department of Clinical and Experimental Medicine, Policlinico “G. Rodolico—San Marco” Hospital, University of Catania, 95123 Catania, Italy; m.ruggieri@unict.it; 3 Department Biomedical and Biotechnological Sciences (BIOMETC), Section of Pharmacology, University of Catania, 95123 Catania, Italygcantare@unict.it (G.C.); bernardi@unict.it (R.B.); 4 Clinical Toxicology Unit, Policlinico “G. Rodolico-San Marco” Hospital, 95123 Catania, Italy; 5 Laboratory of Molecular Biology of Neurometabolic Diseases, Neuroscience Department, Meyer Children’s Hospital IRCCS, 50139 Florence, Italy; anna.caciotti@meyer.it (A.C.); amelia.morrone@unifi.it (A.M.); 6 Department of NEUROFARBA, University of Florence, 50121 Florence, Italy

**Keywords:** Wilson’s disease, ATB7B, obesity, steatotic liver disease

## Abstract

Wilson’s disease (WD) is a biallelic disease-causing variant in the *ATP7B* gene on chromosome 13q14.3 that results in copper accumulation in many organs, particularly the liver and brain. The phenotypic spectrum is wide and symptoms at onset can be heterogeneous. We describe two Sicilian siblings, a young man and his elder sister, both compound heterozygous for the variants c.1286-2A>G and c.2668G>A (p.Val890Met) in the *ATB7B* gene. The male patient presented with liver cirrhosis, which quickly progressed to end-stage liver disease (Child–Pugh score = C10), while his sister had moderate steatotic liver disease (SLD). Our findings highlight that SLD may not always be related to obesity in overweight patients, especially when there are other potential risk factors such as a family history of chronic liver disease, or the persistence of high transaminase despite the adoption of adequate dietary and pharmacological intervention. Screening for conditions such as WD could identify patients at risk of developing SLD and avoid delays in diagnosis. Phenotypic variability in WD is considerable; therefore, further studies are needed to identify which WD patients have a greater risk of developing SLD and determine factors that can predict the severity of the disease.

## 1. Introduction

Wilson’s disease (WD) is a disorder of copper metabolism caused by pathological copper (Cu) accumulation in many organs, particularly the liver and brain. WD is due to homozygous or compound heterozygous pathogenic variants in the ATP7B gene, which encodes a transmembrane Cu-transporting ATPase that is expressed mainly in liver cells. ATP7B protein facilitates transmembrane transport of copper within hepatocytes. ATP7B protein absence or impairment leads to a reduction in biliary excretion of copper; therefore, a toxic accumulation of copper in hepatocytes triggers an inflammatory response. If hepatic storage capacity is saturated, an excessive amount of copper could be released into circulation, resulting in secondary pathological accumulation in some tissues, like nervous tissue (subthalamus, putamen, cortex), and a minor amount in the kidney, eyes (cornea), or other tissues [1].

The loss of function of ATP7B is also responsible for reduced ceruloplasmin blood levels and half-life.

WD is one of the few genetic disorders that can be successfully managed if diagnosed early and correctly treated; however, if left untreated, WD could severely impair liver function and conduct affected people to a condition of fatal acute liver failure or acute-on-chronic liver failure (ACLF) [2].

The incidence of WD is one in 3000 people worldwide, although prevalence differs by geographical region and seems to be higher in the Asian population than in the Caucasian population. The highest disease rates are found in the populations of Costa Rica (4.9/100,000 inhabitants), Germany (2.5/100,000 inhabitants), Japan (3.3/100,000 inhabitants), and Austria [3,4].

In Italy, the highest incidence of WD is in Sardinia (370 per million births), where there is likely to be a founder effect. Six disease-causing variants account for around 85% of ATP7B variants identified [5]. The main clinical manifestations of WD are usually hepatic, with elevation of liver cytolysis enzymes (aspartate aminotransferase; AST and alanine transaminase; ALT) or abnormal findings on liver ultrasound (US). Once the disease progresses, patients may present signs and symptoms of chronic liver disease (e.g., hepatosplenomegaly), or complications due to cirrhotic evolution (e.g., ascites and esophageal varices bleeding).

Neurological symptoms may also appear and include incoordination (e.g., handwriting deterioration—dysgraphia), language difficulties (dysarthria, dyslalia), or movement disorders (e.g., tremors), with subsequent declining performance at school. Mild cognitive impairment, for example in working memory, is also reported [6].

Psychiatric disorders are also frequent and include behavioral and personality problems (aggression and impulsivity), mood disorders (depression, anxiety, and bipolar), and sometimes psychosis [2,7]. Other multisystem involvement can include eye disorders (Kayser–Fleischer ring caused by copper deposition in the cornea), hemolytic anemia and thrombocytopenia, renal tubular acidosis, aminoaciduria, and endocrine (growth and puberty disorders, hypoparathyroidism, dysmenorrhea, and infertility) and cardiac systems (electrocardiography changes and diastolic dysfunction) [8].

WD is one of the major causes in children or young people of hypertransaminasemia and/or neuro-psychic disorders; it should be excluded in this population.

Routine laboratory tests for the diagnosis of WD are serum ceruloplasmin (decreased by 50% of lower normal value), serum free copper (>1.6 µmol/L), 24 h urinary copper (>0.64 µmol/24 h in children; >1.6 µmol/24 h). They represent a screening method to hypothesize a copper disorder and are easily reproducible, not particularly expensive, and accessible.

Diagnostic sensitivity and specificity are not absolute, and other physiological or pathological conditions should be taken into count, such as pregnancy or hormonal treatments, hepatic diseases (autoimmune hepatitis, severe hepatic insufficiency, advanced liver diseases, coeliac disease, familiar aceruloplasminemia), cholestatic syndromes, malabsorption, immunologic assays, incorrect collection of the sample, contamination.

For this reason, they are not sufficient to meet diagnosis, and according to the “European Association for the study of the Liver” (EASL) clinical practice guidelines, a diagnostic score proposed by “the working Party at the 8th international meeting on Wilson Disease, Leipzig 2001” was based also on other features. They are hepatic copper staining on biopsy (but a limit is due to the regional variation), a Kayser–Fleischer ring by slit lamp examination (but it is often absent in up to 50% of patients with WD), neurological symptoms, radiological imaging of the brain findings (e.g., the face of the giant panda sign), Coombs-negative hemolytic anemia, and genetic testing.

Genetics represents the most sensitive diagnostic test, and often it is helpful in cases wherein clinical or laboratory findings are insufficient to make a diagnosis or to facilitate familial screening after diagnosis.

Early diagnosis and treatment modify the natural history of WD. Treatment is life-long and it is influenced by adherence and nutrition indications. D-penicillamine, trientine, and zinc are the most used drugs available for the treatment of WD. Their efficacy is based on chelation through the promotion of urinary excretion of copper or the reduction in intestinal absorption through the induction of the synthesis of metallothioneins in enterocytes [9].

Precise correlations between genotypes and phenotypes in WD have not yet been established. Factors other than genetic ones (environmental, epigenetic) may be responsible for the clinical heterogeneity of WD patients. Moreover, obesity and excess weight may represent “confounding” factors in the presence of steatotic liver disease (SLD), thus delaying the diagnosis of WD.

We hereby report two siblings affected by WD with very different phenotypes, in which the presence of obesity may have delayed diagnosis.

## 2. Case Report

We report two patients, a brother and sister with episodes of hypertransaminasemia in early youth, who were not accurately diagnosed until the third decade of life.

The parents are non-consanguineous, the mother of Italian origin and the father of Tunisian origin.

Familiar medical history was negative for hepatic or nervous diseases with the exception of the paternal grandmother who died with liver cirrhosis of unknown etiology.

All family members (parents and siblings: four people) underwent genetic testing for WD.

### 2.1. Patient n°1

The first patient is a 22-year-old man, second-born from unrelated parents, after a normal pregnancy and natural childbirth. His psychomotor development was normal and his clinical history in the first years of life was unremarkable.

Various episodes of hypertransaminasemia were reported at the age of 4 years, without further diagnostic investigations.

The patient was seen for the first time at the Clinical Hepatology Department of University Hospital “Policlinico-G. Rodolico” in Catania, Italy, in March 2023.

A first physical examination reveals Obesity Class II (body mass index, BMI = 33), splenomegaly, and hepatomegaly.

Blood tests (as reported in Table 1) showed thrombocytopenia (Patient n°1) and altered liver cytolysis and cholestatic parameters.

An abdomen ultrasound showed an enlarged liver with irregular and cocooned margins and a non-homogeneous ultrasound structure.

Because WD was suspected, plasma ceruloplasmin and serum copper were measured. Both were lower than normal. Urine collection over 24 h showed an increase in urinary copper excretion (458 μg/L–n.v. 5–60 μg/L).

An eye test with fundus oculi examination found KF rings, consistent with WD (Leipzig score 6).

**Figure 1:**
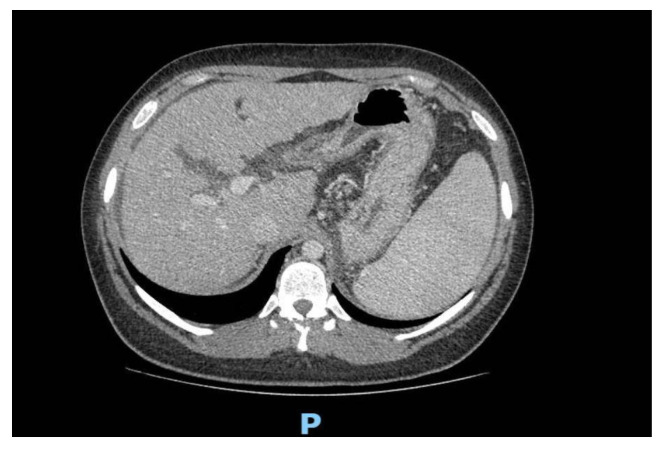
Abdominal computed tomography of Patient n°1. Many cirrhotic nodules are evident as hyper-density areas in the enlarged liver. LEGEND: P = posterior.

Abdominal computed tomography (CT) showed an inhomogeneous structure of the liver, enlarged caudate lobe, blunt margins, enlarged spleen, and ectatic portal vein (17 mm) (Figure 1).

**Figure 2:**
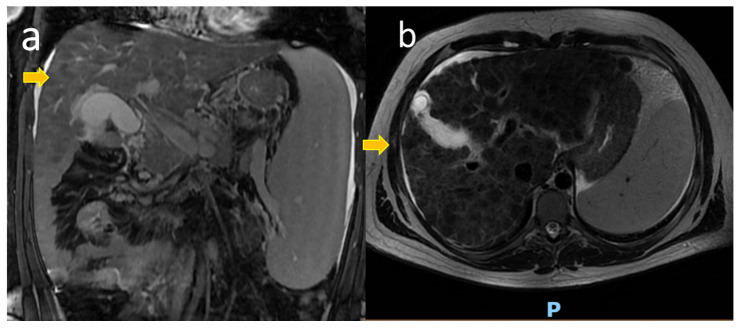
Upper abdominal Magnetic Resonance Imaging (MRI) of Patient n°1: (**a**) Coronal T2 weighted section; (**b**) Axial T2 weighted section; diffusely hyperintense hepatic nodules are evident, as indicated by the arrows. LEGEND: P = posterior.

Upper abdominal Magnetic Resonance Imaging (MRI) showed diffusely hyperintense hepatic nodules as when in accumulation of metals (Figure 2).

Because of liver cirrhosis an esophagogastroduodenoscopy (EGD) was performed and showed esophageal varices grade F1.

Brain MRI was normal and excluded nervous system involvement.

Chelating therapy with D-penicillamine (1500 mg/d; 20 mg/kg/d) was immediately started, according to recent guidelines [10]. After a few weeks, liver function indices (especially transaminases) improved and eventually normalize once the optimal dosage of penicillamine was reached. However, but due to the advanced stage of cirrhosis, the patient was sent to a transplant center.

### 2.2. Patient n°2

The second patient was the older sister of Patient n°1, aged 24 years old. She was the first-born child, delivered after a normal pregnancy, by natural childbirth. Her psychomotor development was normal but her clinical history in the first years of life was characterized by the presence of congenital hydrocephalus, for which she regularly saw a neurosurgery specialist.

At the age of 6 years, she had various episodes of hypertransaminasemia, without diagnostic investigations being carried out. Her personal history showed type 2 diabetes mellitus under treatment with metformin.

WD was suspected after the diagnosis of her younger brother. Ceruloplasmin and serum copper levels were both lower than normal values, with an increase in urinary copper excretion (310 μg/L–n.v. 5–60 μg/L).

At the first physical examination, Obesity Class II (BMI 35) was reported. Abdomen ultrasound described mild hepatic steatosis. Brain MRI did not show any sign suggestive of WD.

Shear Wave Liver Elastography was 4.5 kPa, IQR: 0.7 kPa, IQR/median: 15.6%, (F0–F1).

Eye test with fundus oculi examination did not detect KF rings.

We decided to continue follow-up (haemato-chemical tests of liver function, abdomen ultrasound, and Shear Wave Liver Elastography).

Given the clinical features and diagnostic tests of both the siblings and considering the fast evolution in liver cirrhosis of Patient n°1, a chelating treatment with D-penicillamine was also started in Patient n°2.

Genetic tests for the WD disease-causing variants revealed the same ATB7B genotype found in the patient’s brother (c.1286-2A>G and c.2668G>A) (Table 2).

The c.1286-2A>G variant was identified in a heterozygous state in the ATP7B gene of both siblings and was found to be inherited from the mother. This splice-site pathogenic variant occurs in the acceptor splice site of exon 3, and it is predicted to remove the Cu5 binding domain, affecting protein folding and the interaction of ATP7B with the ATOX-1 chaperone [11]. The variant has previously been reported and involves a canonical splicing site in the ATP7B gene.

The c.2668G>A (p.Val890Met) variant has been previously reported [12], and it was inherited from the patients’ father. This sequence change replaces valine, which is a neutral and non-polar amino acid, with methionine, which is neutral and non-polar, at codon 890 of the ATP7B protein (p.Val890Met). Advanced modeling of the protein sequence and biophysical properties indicated that this missense variant is expected to disrupt the ATP7B protein function. All patients’ details are summarized below (Table 3).

**Table 3:** Genotype–phenotype comparison between the two patients in our study.

	Patient n°1	Patient n°2
**Sex**	M	F
**Type of Onset**	Hepatic	Hepatic
**Genotype**	c.1286-2A>G/c.2668G>A (p.Val890Met)	c.1286-2A>G/c.2668G>A (p.Val890Met)
**Age of Onset**	4	6
**Age of Diagnosis**	22	24
**Physical Examination**	Obesity Class II (BMI 33), splenomegaly and hepatomegaly	Obesity Class II (BMI 35)
**Blood Tests**	Low ceruloplasmin and copper, thrombocytopenia, hypertransaminasemia (see Table 1)	Low ceruloplasmin and copper (see Table 1)
**Eye Examination**	KF rings in both eyes	No KF rings
**Brain MRI**	No obvious signs of involvement from WD	Absence of pathological signs of WD
**Leipzig Score (before Genetic Test)**	6	4
**Abdominal US**	Normal liver volume with irregular and cocooned margins and a non-homogeneous echostructure. Hypoechoic nodular formation in the 7th segment of 15 mm. Splenomegaly	Mild hepatic steatosis

LEGEND: BMI = Body Mass Index; KF = Kayser–Fleischer.

## 3. Discussion

WD is a biallelic genetic disorder characterized by a wide phenotypic variability caused by copper accumulation in various organs; hepatic and/or neurological involvement frequently occur in WD patients. The broad spectrum of liver disease ranges from asymptomatic cases (with only elevated transaminases) to steatosis, cirrhosis, and acute liver failure (ALF). Although copper accumulation starts at birth, WD symptoms usually appear later in life, mainly in the second to the fourth decade. Timely detection and appropriate treatment usually result in the resolution of symptoms and an improved quality of life.

Consequently, genetic testing of family members screening is very important for identifying asymptomatic patients before clinical onset. Indeed, liver and brain organ damage may be present even in asymptomatic patients, making timely identification critical.

Precise genotype–phenotype correlations have not yet been proven, and other potential modifying factors are likely to influence the severity and outcome of WD [1].

Several reports have shown significant differences in genotype–phenotype correlations, which suggests that epigenetic/environmental factors have a significant impact on WD phenotypes [13].

Although the here-reported siblings share the same ATP7B genetic variants, their WD phenotypes were quite different. The brother presented liver cirrhosis without neurological signs at onset, and he began therapy before genetic testing, as his Leipzig score of > 4 confirmed the diagnosis. His sister also had no neurological involvement related to WD (her hydrocephalus was congenital), but she presented hepatic steatosis revealed by US. Her Leipzig score was 4, so a genetic test was necessary before starting therapy. In accordance with the EASL guidelines and due to significant hepatic involvement [9], her brother began chelation treatment with D-penicillamine.

Therefore, both siblings showed hepatic involvement at the onset of WD, albeit of different severity, and without neurological symptoms. The pathogenic variant c.2668G>A (p.Val890Met) has been described before in the literature in children with hepatic onset of WD, also in cases with acute liver failure, both in homozygosity and in compound heterozygosity [14,15], as in our siblings, suggesting a potential genotype–phenotype correlation.

However, phenotypic variability in WD is considerable, and it is not yet clear whether other genetic and/or environmental factors contributed to the different severity of hepatic WD in these siblings.

The presence of obesity at the onset of WD can be interpreted in several ways. On the one hand, the increased prevalence of obesity in developed countries will present hepatology centers with an increasing number of cases of SLD associated with obesity. The development of obesity can be influenced by many factors, including diet, physical activity, and lifestyle choices. A child affected by WD, but not yet diagnosed, may manifest obesity and SLD, also accompanied by hypertransaminasemia, as a result of the influence of environmental factors, rather than the WD itself, due to the young age and relatively “short” disease duration, with limited liver damage compared to an adult naïve WD patient with hepatic involvement. In this case, an adequate modification of the lifestyle, with frequent periodic follow-ups, is required to verify if this is sufficient to reduce obesity and, above all, the SLD. The persistence of SLD despite appropriate lifestyle interventions constitutes a further suspicion factor for WD in childhood. Therefore, if an adult WD patient, never diagnosed, develops obesity, SLD could be due to a combination of environmental factors and the disease progression itself.

In this regard, our case demonstrates that SLD may not be caused only by co-existing obesity, especially when other factors are present (young age, family history of chronic liver disease, persistence of high transaminase values despite appropriate dietary and pharmacological measures). In such cases, screening for possible causes, including WD, may be crucial.

Hepatic steatosis in WD is probably caused by copper accumulation in the liver tissue, but it is possible that metabolic changes, such as those triggered by obesity, play a role in the pathogenesis of steatosis in these WD patients [16]. Diabetes and insulin resistance have also occasionally been reported in WD patients [17]. Excessive fat deposition in the liver and nuclear glycogen deposition have been proposed as contributing factors to hepatic insulin resistance in these individuals. However, hepatic macrosteatosis and glycogenated nuclei, which are also characteristics of SLD, are the most commonly detected hepatic histological lesions in WD [18].

The patient in our case had significant obesity and an increase in transaminases, which could be cause for concern and may require further hepatic investigations. During his childhood, he experienced unexplained hypertransaminasemia. Unfortunately, appropriate investigations were not conducted at that time, and WD was initially disregarded in our patient because of his SLD and excessive obesity, leading to a 20-year diagnostic delay.

Our case highlights the significance of early and appropriate investigations since childhood, as WD is still a genetic disease that is burdened by diagnostic delay. Further studies are needed to identify which WD patients have a greater risk of developing SLD and determine factors that can predict the severity of the disease.

## Figures and Tables

**Table 1:** Blood tests of two patients.

Blood Test	Patient N°1	Patient N°2	Normal Value (n.v.)
Ceruloplasmin (mg/dL)	5.1	7	22–58
Serum copper (μg/dL)	18	124	70–140
Urinary copper in 24 h (μg/L)	458	310	5–60
Hb (g/dL)	14.1	12.4	12–16
RBC (mmc)	4,340,000	4,620,000	3,800,000–5,500,000
MCV (fL)	88	82,5	80–97
WBC (mmc)	5710	7250	4000–10,000
PLT (mmc)	112,000	300,000	140,000–400,000
Total bilirubin (mg/dL)	0.66	NA	0.01–1
Direct bilirubin (mg/dL)	0.43	NA	0.01–0.3
Total proteins (g/dL)	6.5	NA	6–8.5
Albumin (g/dL)	3.7	3.77	4.02–4.76
INR	1.62	NA	0.8–1.2
ALT (U/L)	110	63	2–49
AST (U/L)	140	47	40–145
ALP (U/L)	177	NA	30–120
GGT (U/L)	194	NA	0–38
Cholesterol (mg/dL)	161	162	<200
Cholesterol HDL (mg/dL)	40	NA	>60
Cholesterol LDL (mg/dL)	91	NA	80–130
Triglycerides (mg/dL)	152	203	0–150

LEGEND: Hb = hemoglobin; RBC = red blood cells; WBC = white blood cells; MCV = mean corpuscular volume; PLT = platelets; INR = international normalized ratio; ALT = alanine aminotransferase; AST = aspartate transaminase; ALP = alkaline phosphatase; GGT = gamma-glutamyl transferase; HDL = high-density lipoprotein; LDL = low-density lipoprotein; NA = not available.

**Table 2:** Variants identified in Patient n°1 and Patient n°2.

Gene	Transcript	Phenotypes Associated	Transmission Model	Variant	State	HGMD	Frequency in gnomAD (Alleles)	Classification ACMG
ATP7B	NM_000053	Wilson’s disease (OMIM #277900)	BA	c.1286-2A>G	Mat heterozygosity	Absent	Absent	Likely pathogenic
ATP7B	NM_000053	Wilson’s disease (OMIM #277900)	BA	c.2668G>A (p.Val890Met)	Pat heterozygosity	Pathogenic	5/280992 (no homozygous individuals)	Likely pathogenic

LEGEND: HGMD: Human Gene Mutation Database, gnomAD: Genome Aggregation Database, ACMG: American College of Medical Genetics and Genomics, BA: biallelic, pat: paternal, mat: maternal.

## Data Availability

The data presented in this study are available on request from the corresponding author.
